# Lipidomics of Bioactive Lipids in Acute Coronary Syndromes

**DOI:** 10.3390/ijms20051051

**Published:** 2019-02-28

**Authors:** Zahra Solati, Amir Ravandi

**Affiliations:** 1 Institute of Cardiovascular Sciences, St. Boniface Hospital Research Centre, University of Manitoba, Winnipeg, MB R2H 2A6, Canada; solatiz@myumanitoba.ca; 2 Department of Physiology and Pathophysiology, University of Manitoba, Winnipeg, MB R3E 3P5, Canada; 3 Section of Cardiology, Department of Internal Medicine, Max Rady College of Medicine, Faculty of Health Sciences, University of Manitoba, 409 Tache Avenue, Winnipeg, MB R2H 2A6, Canada

**Keywords:** oxidized phospholipids, oxylipins, bioactive lipids, coronary disease, myocardial infarction, ischemic heart disease, ischemia reperfusion injury, mass spectrometry, lipids

## Abstract

Acute coronary syndrome (ACS) refers to ischemic conditions that occur as a result of atherosclerotic plaque rupture and thrombus formation. It has been shown that lipid peroxidation may cause plaque instability by inducing inflammation, apoptosis, and neovascularization. There is some evidence showing that these oxidized lipids may have a prognostic value in ACS. For instance, higher levels of oxidized phospholipids on apo B-100 lipoproteins (OxPL/apoB) predicted cardiovascular events independent of traditional risk factors, C-reactive protein (hsCRP), and the Framingham Risk Score (FRS). A recent cross-sectional study showed that levels of oxylipins, namely 8,9-DiHETrE and 16-HETE, were significantly associated with cardiovascular and cerebrovascular events, respectively. They found that with every 1 nmol/L increase in the concentrations of 8,9-DiHETrE, the odds of ACS increased by 454-fold. As lipid peroxidation makes heterogonous pools of secondary products, therefore, rapid multi-analyte quantification methods are needed for their assessment. Conventional lipid assessment methods such as chemical reagents or immunoassays lack specificity and sensitivity. Lipidomics may provide another layer of a detailed molecular level to lipid assessment, which may eventually lead to exploring novel biomarkers and/or new treatment options. Here, we will briefly review the lipidomics of bioactive lipids in ACS.

## 1. Introduction

Acute coronary syndrome (ACS) comprises a set of ischemic conditions including unstable angina (UA), myocardial infarction (MI) (with or without ST-segment elevation), and sudden cardiac death. It is the most common cause of morbidity and mortality worldwide, and accounts for roughly seven million deaths and 129 million loss of disability-adjusted life years (DALYs) annually [1]. The main cause of ischemia is the reduction of blood flow into coronary microcirculation as a result of atherosclerotic plaque rupture and thrombus formation [2]. Complete occlusion of coronary arteries usually presents with ST-segment elevation myocardial infarction (STEMI), which is accompanied by tissue injury and presents with elevated troponin levels. Partially occluded coronary arteries may result in non-STEMI or UA, depending on whether or not myocardial injury occurs [3]. 

Coronary angiography has shown that the atherosclerosis extent index (including the number of diseased vessels, stenosis and occlusions) is generally lower in ACS patients than in patients with stable angina, suggesting that plaque vulnerability rather than the extent of atherosclerosis may be the determinant of ACS [4]. The mechanisms leading to the progression of an asymptomatic plaque to a vulnerable one are not fully understood. A thin fibrous cap and a large lipid core (≥40% plaque volume), inflammatory cells, and high neovascularity are suggested as factors causing plaque vulnerability [5]. 

The oxidation of lipoproteins, namely oxidized low-density lipoproteins (Ox-LDLs), has been considered as a key factor in this transition through various mechanisms. Following the infiltration of LDL into the injured endothelium, LDL becomes oxidized to form Ox-LDL. This modified LDL elevates the expression of cell adhesion molecules such as intercellular cell adhesion molecule-1 (ICAM-1) and vascular cell adhesion molecule-1 (VCAM-1), resulting in leukocyte (mainly monocytes and T-lymphocytes) recruitment and migration into the intima. In the intima, monocytes differentiate into macrophages. These lipid laden macrophages, which are called foam cells, along with the migrated T-lymphocytes release a variety of cytokines that promote inflammation and the generation of reactive oxygen species (ROS) [6]. Ox-LDL increases the infiltration of macrophages into the plaque (foam cell formation), up-regulates the expression of matrix metalloproteinase (MMP), and triggers proinflammatory reactions leading to plaque rupture [7].

Several clinical studies have confirmed that Ox-LDL concentrations are significantly higher in MI patients when compared with stable angina or age-matched controls [8,9,10]. Lipid peroxidation can occur within the LDL membrane through non-enzymatic and/or enzymatic mechanisms, producing diverse secondary products such as 4-hydroxynonenal (4-HNE), malondialdehyde (MDA), oxidized phospholipids (OxPLs), and oxylipins. These oxidized lipids are bioactive and can be bound to proteins, peptides, phospholipids, and nucleic acids, generating structural neo-epitopes called oxidation-specific epitopes (OSEs). Consequently, chronic elevations of OSEs may induce inflammation through the secretion of chemokines and proinflammatory cytokines, leading to plaque instability [11]. Clinical studies have also confirmed higher levels of these bioactive molecules in ACS patients when compared with patients in control groups [12,13,14,15]. 

Previous studies have shown that bioactive lipids can predict ACS occurrence in various populations. For instance, higher levels of OxPLs on Ox-LDL have been found to predict the progression of first or second major coronary events [16,17]. In addition, a recent cross-sectional study showed that levels of oxylipins, namely dihydroxy-eicosatrienoic acid (DiHETrE) and 16- hydroxy-eicosatetraenoic acid (HETE), were significantly associated with cardiovascular and cerebrovascular events, respectively. In this study, levels of 8,9-DiHETrE were significantly higher in patients with ACS (*n* = 24) compared to those without ACS (*n* = 74). Univariate and multivariate logistic regression also revealed that 8,9-DiHETrE concentrations were significantly associated with the presence of ACS. Moreover, they found that with every 1 nmol/L increase in the 8,9-DiHETrE concentrations, the odds of ACS increased by 454-fold. In this particular study, 8,9-DiHETrE elevated the odds of ACS by 92-fold [18].

Bioactive lipids have been measured conventionally by the use of chemical reagents, immunoassays, or chromatography [19]; however, these methods have limitations such as the lack of sensitivity and specificity. The main drawback of using conventional methods is that only one analyte can be assessed with one set of analysis. Considering the heterogeneity of pools of oxidized lipids, rapid multi-analyte quantification methods are needed. With the advent of robust mass spectrometric techniques, various groups of compounds can be assessed at the same time in a targeted and non-targeted fashion. By using soft ionization mass spectrometry (MS) such as electrospray ionization (ESI), the identification and quantification of non-volatile and thermolabile samples such as OxPL and oxylipins are feasible. Lipidomics is a powerful tool providing another layer of the detailed molecular levels of lipid assessments that may help to explore novel biomarkers and new treatment options in ACS [20]. 

In this article, we will briefly review the mechanisms in which bioactive lipids are generated. Then, we will focus on the analytical methods used by previous studies to measure these compounds. Finally, we will review the clinical studies that have assessed the roles of bioactive lipids in ACS patients. 

## 2. Bioactive Lipid Generation

**Figure 1:**
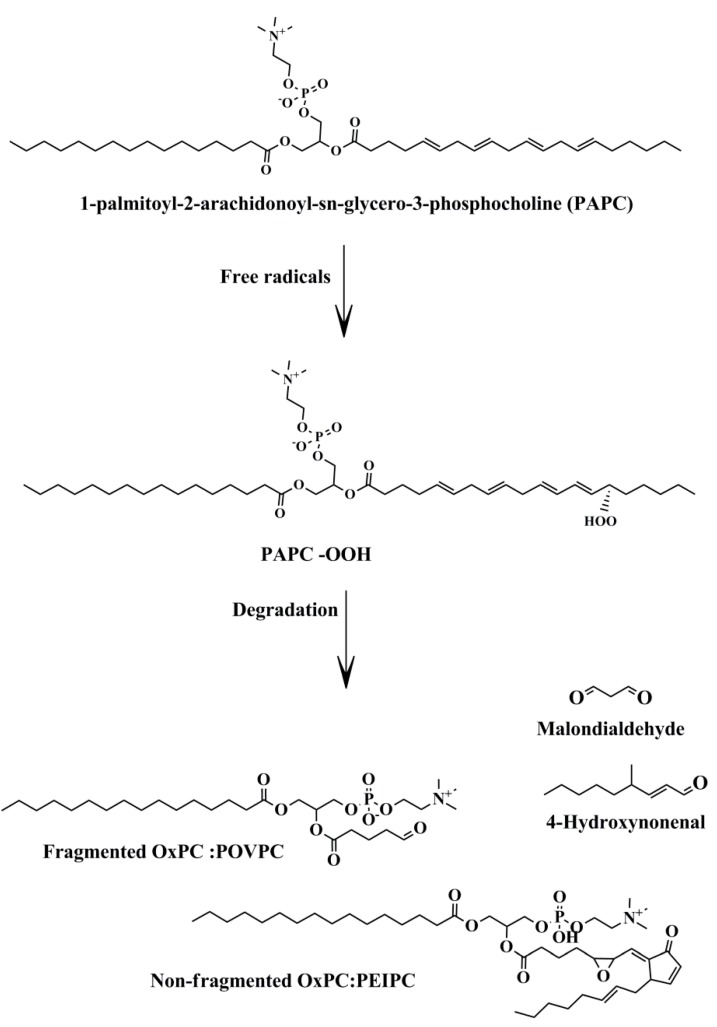
Non-enzymatic oxidation of membrane phospholipids. Free radicals may attack membrane phospholipids such as PAPC, leading to the production of bioactive lipid molecules. Abbreviations: PAPC-OOH, PAPC hydroproxide; OxPC, oxidized phosphatidylcholine; PEIPC, 1-palmitoyl-2-(5,6-epoxyisoprostane E2)-sn-glycero-3-phosphocholine.

About 700 phospholipid (PL) molecules have been identified on the surface of LDL particles [6]. Phosphatidylcholine (PC) and sphingomyelin (SM) are the main PLs in LDL particles [21]. Most PLs contain polyunsaturated fatty acids (PUFAs), with 14–24 carbons in their sn-2 position, which make them susceptible to oxidation. They can undergo non-enzymatic oxidation mainly by ROS, making heterogeneous pools of oxidized lipids. Hydroperoxides (LOOH) are the first products of PUFA oxidation by ROS. During degradation of LOOH, a large variety of secondary products are produced such as 4-hydroxynonenal (4-HNE), malondialdehyde (MDA), non-fragmented (full length), and fragmented (shorten chain) OxPLs [22] (Figure 1).

4-HNE is a α,β-hydroxyalkenal which is formed through the peroxidation of arachidonic acid (AA) (20-carbon compounds) and linoleic acid (LA) (18-carbon compounds). Its reaction with the histidine, cysteine, or lysine residues of proteins makes Schiff bases or Michael adducts. MDA is a three-carbon aldehyde that is similarly produced through the non-enzymatic oxidation of PUFA. It can also be produced as a side product of thromboxane A2 (TXA2) synthesis. AA and docosahexaenoic acid (DHA) are the main precursors of MDA [23]. Levels of 4-HNE and MDA increase during oxidative stress and have been widely accepted as markers of oxidative stress. 

**Figure 2:**
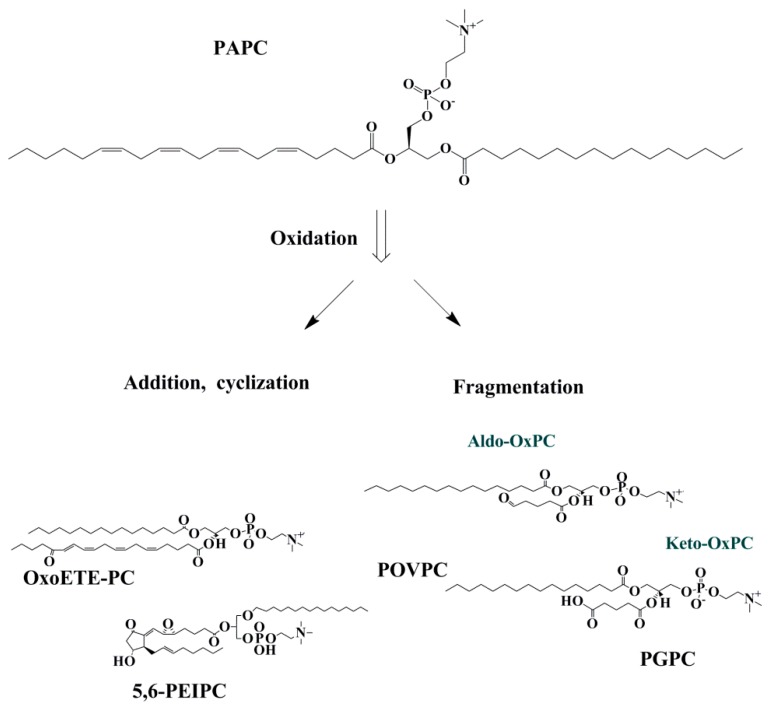
Fragmented and non-fragmented OxPC productions from PAPC. OxPLs can be classified as fragmented and non-fragmented species. Non- fragmented species are produced from the addition of peroxyl radicals where rearrangement/cyclization may happen. Fragmented species are comprised of aldehyde and carboxylic acid containing lipids. Abbreviations: Oxo-ETE-PC, oxoeicosatetraenoic acid phosphocholine; PEIPC, 1-palmitoyl-2-(5,6-epoxyisoprostane E2)-sn-glycero-3-phosphocholine; Aldo-OxPC, aldehyde containing oxidized phosphatidylcholine; Keto OxPC; carboxylic acid containing oxidized phosphatidylcholine; POVPC, 1-palmitoyl-2-(5′-oxo-valeroyl)-sn-glycero-3-phosphocholine; PGPC, 1-palmitoyl-2-glutaryl-sn-glycero-3-phosphocholine.

OxPLs can be divided into two groups of non-fragmented (with the same number of carbon with precursor) and fragmented (with shorter chain) OxPLs. Non-fragmented OxPLs are formed following the initial phase of lipid oxidation. Then, they may undergo intramolecular cyclization, rearrangement, and further oxidation and make OxPLs with terminal furans, isoprostanes, and long-chain products with functional groups such as hydroperoxides, hydroxides, keto- and epoxy-groups [24]. Fragmented OxPLs have hydroxide and carbonyl groups in their structures, which are highly bioactive and can rapidly interact with biomolecules causing tissue injury [25] (Figure 2). 

**Figure 3:**
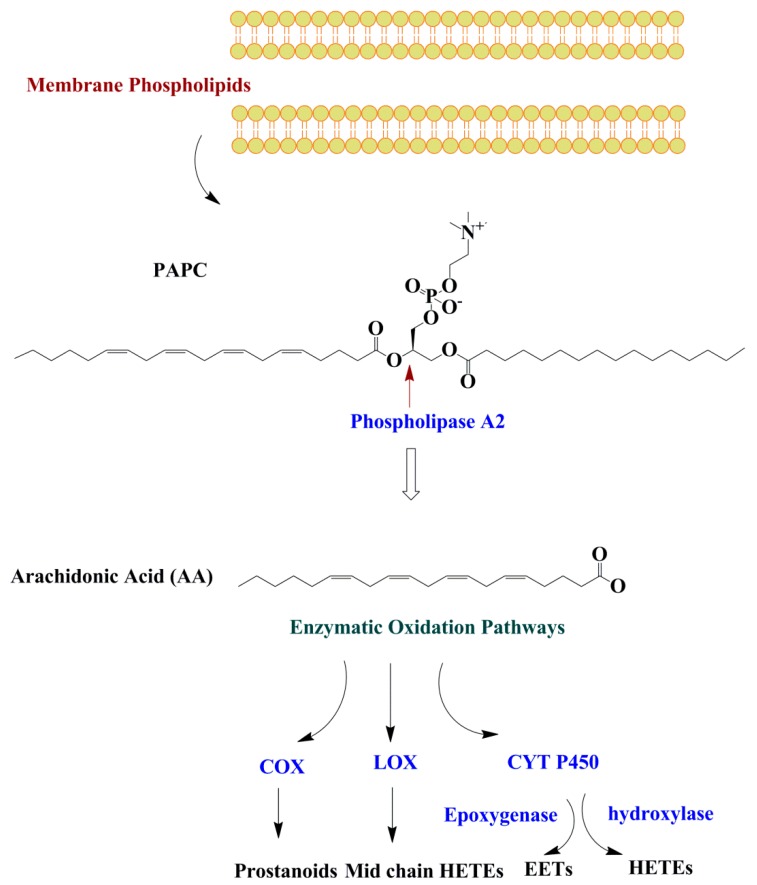
Enzymatic oxidation of membrane phospholipids. Fatty acids are released from the membrane PL by the phospholipase A2 enzyme and may undergo oxidation through three oxidation pathways including COX, LOX, and CYT P450. Abbreviations: COX, cyclooxygenase; LOX, lipoxygenase; CTY P450, cytochrome P450; HETE; hydroxyeicosatetraenoic acids; EET, epoxyeicosatrienoic acids.

All PUFAs including omega-3 PUFAs are oxidized by the three main enzymes of cyclooxygenase (COX), lipoxygenase (LOX), and cytochrome P450 (CYP). The types of oxylipins produced from the PUFAs depend on the type/amounts of dietary PUFA, and the availability and affinity of the enzymes (COX, LOX, or CYP) for a specific substrate PUFA. The most well-known oxylipins are derived from AA and LA [1]. Half of the known oxylipins are derived from AA. However, other oxylipins can also be produced from PUFAs besides AA including both the omega-3 and omega-6 PUFA. It is important to mention that phospholipase-A2 (PL-A2), which has a key role in oxylipin production, has a preference for AA and eicosapentaenoic acid (EPA) [26]. These fatty acids may undergo enzymatic oxidation through cyclooxygenase (COX), lipoxygenase (LOX), and cytochrome P450 (CYP) pathways. Oxylipins are not stored in the cells and exert their biological roles through paracrine or autocrine mechanisms [27] before they are chemically inactivated or re-esterified into a glycerolipid pool [28] (Figure 3). 

Prostanoids (prostaglandines (PG) and thromboxanes (TX)) and some forms of hydroxy-metabolites such as 11-HETE are generated through the COX pathway from AA. LOX enzymes catalyze the generation of hydroxy fatty acids such as leukotrienes, lipoxins, resolvins, protectins, maresins, hepoxilins, and eoxins [3,29]. Mid chain (5-, 8-, 9-, 11-, 12-, and 15-) HETEs are also formed from AA through the LOX pathway [18,30]. CYP 450 enzymes have epoxygenase or ω-hydroxylase activity [29]. ω-terminal (16-, 17-, 18-, 19-, and 20-) HETEs are produced from AA and by ω-hydroxylase enzymes (CYP4A and CYP4F) and epoxyeicosatrienoic acid (EETs) are generated by CYPs with epoxygenase activity [28]. 

## 3. Measurement of Bioactive Lipids

### 3.1. 4-HNE and MDA

Free aldehydes can be identified and quantified by several analytical methods. Thiobarbituric acid reactive substance (TBARS)/spectrophotometry has been widely employed to measure MDA levels. Under acidic conditions and high temperatures, the aldehyde group of MDA reacts with the nucleophilic center of TBA and makes a red-colored derivative, which can be detected by spectrophotometric and spectrofluorometric approaches. The aldehyde group of HNE can also make derivatives with 2,4-dinitrophenylhydrazine (DNPH) that are detectable by spectrophotometry [12,14,31,32]. 

Kamiński et al. (2008) measured the HNE and MDA in the plasma of 15 STEMI patients and 10 patients with stable IHD as the control group by using derivatization/high performance lipid chromatography (HPLC)-fluorescence detection [33]. Solid phase extraction was applied to extract HNE and MDA [34]. MDA was also detected using the TBARS derivatization, and then separated and quantified by HPLC-spectrofluorometric assay [35]. 

Gas chromatography (GC) is the other main analytical method to measure MDA and HNE. MS is more specific and sensitive compared with other analytical methods as it can identify these aldehydes based on the mass to charge ratio and fragmentation pattern [36]. GC can also be coupled to MS. Tsikas et al. (2017) developed a method to measure the plasma concentrations of both MDA and HNE simultaneously by using GC/MS. They used pentafluorobenzyl hydroxylamine as a derivatization reagent, [1,3-2H2]-MDA (d2-MDA), and [9,9,9-2H3]-HNE (d3-HNE) as the internal standards. The ionization technique used here was hard ionization with high energy such as electron impact [37] and is different from soft ionization, which will be discussed later.

Syslová et al. (2009) developed a method using reverse phase HPLC/ESI-MS to assess the MDA and HNE in plasma, urine, and exhaled breath condensate [38]. HNE-d3 and Me-MDA was used as the internal standards, butylated hydroxytoluene (BHT) as the antioxidant, and acetonitrile was added to the plasma. Then, the plasma was sonicated and centrifuged to remove the precipitated proteins. The supernatant was dried under nitrogen gas, and re-suspended in acetonitrile to be injected into HPLC. HPLC with a Hypercarb Thermo 100 mm × 2.1 mm × 5 mm column and Hypercarb-precolumn was used. Water and ammonium hydroxide were used as solvent A, and methanol:acetonitrile with ammonium hydroxide was used as the co-solvent. Derivatization with 4-2-trimethylammonio ethoxy benzenaminiumhalide (4-APC) or cyclohexanedione (CHD) can be also done prior to extraction to increase the ionization of these aldehydes [39,40]. 

### 3.2. OxPL

Using monoclonal antibodies is one of the well-established methods to assess the OxPL levels on apoB100-containing lipoproteins, namely LDL, very low-density (VLDL), and lipoprotein (a) (LP (a)). To perform this assay, the murine monoclonal antibody MB47 must be added initially to capture all apoB-100 lipoproteins from the plasma. Then, by adding the E06 antibody, it can bind to apoB-100. This method has been applied extensively to measure the OxPL levels in CVD [41]. The limitation of this method is that only the OxPL species that are present on the apoB100 lipoproteins can be assessed, and not the total amount of OxPL in the plasma. In addition, this method cannot identify specific OxPL species among all types of OxPL (fragmented, non-fragmented OxPL) that are produced during lipid oxidation [19]. To overcome these limitations, LC/MS has been introduced as the best option for a detailed analysis of OxPLs. 

Hassanaly et al. (2017) measured the levels of oxidized phosphatidylinositol (OxPI) in Ox-LDL and human atherosclerotic plaque by using reversed-phase HPLC/ESI-MS. Using this approach, they were able to identify and quantify 23 OxPI species in human Ox-LDL and atherosclerotic plaque. They found that levels of OxPI species increased significantly in Ox-LDL at 48 h when compared with the baseline. Moreover, non-fragmented hydroperoxides were the dominant species in Ox-LDL at 48 h, comprising 52.07% of the total OxPI species. Fragmented aldehyde and carboxylic acid containing OxPI comprised 17.32% and 0.89% of total the OxPI at the same time point. Likewise, in human atherosclerotic plaques, which were retrieved from patients who underwent saphenous vein graft (SVG) interventions, non-fragmented hydroperoxides were the most abundant OxPI compounds. Fragmented aldehyde and carboxylic acid containing OxPI comprised 18.6% and 1.5 % of the total OxPI compounds, respectively [42]. 

OxPC species have been identified in patients that have undergone percutaneous coronary and peripheral procedures by using normal phase HPLC/ESI-MS. In this study, the five most abundant OxPCs were in embolized material captured by distal protection filter devices during uncomplicated saphenous vein graft, carotid, renal, and superficial femoral artery interventions. 1-palmitoyl-2-(9-oxo-nonanoyl) PC (PONPC) was the most abundant fragmented OxPC, which comprised 50% of the total quantified fragmented OxPC compounds. POVPC, 1-palmitoyl-2-glutaroyl-sn-glycero-3-phosphocholine (PGPC), and 1-palmitoyl-2-(5-keto-6-octene-dioyl) PC (KOdiAPC) were the other fragmented OxPC species measured in this study [43]. 

Recently, we were able to identify and quantify 56 OxPC species including both fragmented and non-fragmented OxPCs in rat kidneys following ischemia/reperfusion (I/R) injury. 1-stearoyl-2-linoleoyl-phosphatidylcholine (SLPC-OH) and 1-palmitoyl-2-azelaoyl-sn-glycero-3-phosphocholine (PAzPC) were the most abundant non-fragmented and fragmented OxPC after I/R, respectively. The total levels of OxPC species (including fragmented and non-fragmented OxPC compounds) increased significantly after both 6 h and 24 h reperfusion when compared with the sham group. Concentrations of fragmented OxPCs were elevated significantly by increasing the time of reperfusion as their levels were significantly higher following 24 h reperfusion when compared to 6 h I/R and sham groups. However, no significant differences were observed between the sham and 6 h I/R groups. Changes in the levels of non-fragmented OxPCs were different to the fragmented compounds. Although the total levels of non-fragmented OxPC elevated significantly in the 6 h I/R group, no differences were observed in the 24 h I/R group. These data pointed to the importance of identifying the specific compounds, and not just the total concentrations of the oxidized species [44]. 

The first step in preparing samples for lipidomic analysis is extracting the lipids from the cell/tissue/plasma. Currently, conventional liquid–liquid extraction has been widely used for the extraction of OxPL [45]. Folch extraction, which uses chloroform/methanol, is one of the most common extraction approaches to extract OxPL. Adding antioxidants such as BHT is recommended to minimize further oxidation [46]. Recently, it has been suggested that enrichment strategies such as using gold nanoparticles (GNP) and anti-Ox-LDL antibodies on plasma samples [47] or lipid extracts [48] may increase the efficacy of OxPC identification. Hinterwirth et al. [47] used GNPS with four different Ox-LDL antibodies, namely the E06, anti-Cu Ox-LDL antibody, anti-MDA-LDL antibody, and anti-carboxymethyllysine-LDL antibody, to increase the detection of OxPC in plasma. Stübiger et al. [48] also reported that using 2-aminobenzoic acid (2-AA) as the reagent with GNP elevated the carbonyl-containing OxPC identification at subnanomolar concentrations, with up to 90% recoveries [49]. 

To separate the OxPLs species, reverse phase HPLC with C8 or C18 columns with either isocratic or gradient elutions has been widely used, although they can also be separated by normal phase HPLC [43]. By using HPLC, OxPL are separated based on polarity and molecular weight before interfacing with the MS, which increases the sensitivity of the assessment [50]. Reverse phase mobile phases are usually a mixture of water, methanol, or acetonitrile. Hexane or isopropanol can also be applied as co-solvents. Ammonium acetate, ammonium formate, or acetic or formic acid may also be added to the solvent to facilitate ionization in MS. There are no detruded internal standards for OxPLs analysis. Non-oxidized PLs and lyso PL (LPL) such as phosphatidylinositol (PI) (31:1) for OxPI analysis and PC (9:0)/LPC(17:0) for OxPC analysis have been used as internal standards [42,44]. These PLs have the same structures and fragmentation patterns and are not produced in the body. Therefore, they can be used as an internal standard to assess the extraction efficacy and instrument response [51]. 

ESI/MS and matrix-assisted laser desorption/ionization (MALDI) are two forms of soft ionization techniques. The soft ionization technique allows for the analysis of non-volatile compounds such as OxPL. ESI can readily interface with HPLC. This is very important when analyzing OxPLs as the levels of these compounds are considerably lower when compared with non-oxidized compounds. Therefore, separation techniques prevent ion suppression, which may occur with high abundant molecular ions. On the other hand, sample preparation is simpler with ESI when compared with MALDI as MALDI needs the co-crystallization of a matrix with the sample, which consequently may affect the quantification of the analytes. MALDI can examine solid state samples and is useful for MS imaging of tissue, while ESI needs tissue extraction as it requires a liquid sample [52]. Some studies have used MALDI to quantify chlorinated PL [53,54]. However, no study has measured the levels of oxidized lipids in tissue using MALDI. In a review by Ana Reis (2017), it was emphasized that MS imaging to assess the distribution of OxPL in tissue is challenging due to the low concentrations of OxPL/PL and the lack of fluorescent probes designed to bind to free OxPL in tissue samples [45].

### 3.3. Oxylipins 

Like other oxidized lipids, traditional analytical methods have been widely used for assessing oxylipins [55]. Miler et al. (1985) developed enzyme-linked immunosorbent assays (ELISA) to assess LTC4, LTB4, 6-keto PGF1 alpha, and TXB2 [56]. The main drawback of this approach is that only one analyte can be targeted with one set of analysis. GC/MS has also been utilized to measure oxylipins, for instance, Tsukamoto et al. (2002) developed a method to measure oxylipins including PG, isoprostane and TXs with GC/MS [57]. Due to the complex sample preparation and thermal decomposition during derivatization, HPLC based methods have been used recently for oxylipin analysis [55].

HPLC/ESI-MS has been utilized to quantify plasma oxylipins in patients [18,58]. For instance, Caligiuri et al. (2017) quantified 39 plasma oxylipins in patients with PAD using HPLC/ESI-MS [18]. Among all the identified/quantified oxylipins, 4 oxylipins were significantly correlated with the presence of cardiovascular/cerebrovascular events. For instance, plasma levels of 8,9 DiHETrE were significantly elevated in patients with ACS when compared with ones without ACS (0.3 ± 0.1 versus 0.2 ± 0.0 nM, respectively). Plasma concentrations of PGE2 were significantly higher in patients with angina when compared with subjects without angina (0.4 ± 0.0 versus 0.3 ± 0.0 nM, respectively). Moreover, they found that 16-HETE, TRX B2, and 11,12- DiHETrE increased the odds of having cardiovascular/cerebrovascular events in this population. 

To prepare samples for lipidomic analysis, oxylipins can be extracted using liquid–liquid extraction and/or solid-phase extraction procedures. Use of chloroform/methanol mixtures, according to Bligh and Dyer, is the most common liquid–liquid extraction protocol for oxylipins. In this method, oxylipins are dissolved in organic solvents, but hydrophilic materials such as proteins are eliminated following phase separation. Solid-phase extraction can be conducted using commercial columns pre-packed with various sorbents. Reverse-phase HPLC with a C18 column has been used widely to separate oxylipins [18]; however, Zu 2016 et al. used ultra-performance liquid chromatography (UPLC) (C18 column) to separate oxylipins before analysis with MS [15]. The UPLC column has better resolution, lower detection limits, and a shorter chromatographic run when compared with HPLC [51]. Deuterated oxylipins are commercially available, which are used as internal standards. These standards are matched with groups of endogenous oxylipin species in terms of chemistry, retention time, and ionization efficiencies [51]; as mentioned previously, internal standards are needed to assess the extraction efficacy and instrument response [51]. 

Tandem mass spectrometry is the most sensitive system for analyzing oxidized lipids, particularly when predetermined species are desired, and is known as the targeted approach. Multiple-reaction monitoring (MRM) is an acquisition mode that monitors the transition of a selected precursor ion, based on the mass/charge value, to a specific product ion using the fragmentation pattern. It has been reported that by using separation techniques such as UPLC and MRM transitions, more than hundreds of oxylipins can be identified/quantified in a single acquisition at picogram/fentomole levels [59]. 

Quantifications of oxidized lipids can be carried out by generating calibration curves for internal standards. As mentioned previously, deuterated oxylipins are commercially available, which can be used as oxylipin internal standards. However, non-oxidized PLs and lyso PL (LPL) such as PI (31:1), PC (9:0), and LPC (17:0) have been used as internal standards for OxPI and OxPC quantifications as there is no deuterated standard for OxPL analysis. 

## 4. Role of Bioactive Lipids in ACS

### 4.1. 4-HNE and MDA

Previous studies have shown that 4-HNE may contribute to many cardiovascular diseases (CVD) [60,61,62]. It can be generated during Ox-LDL oxidation and makes apo B-adducts, which are identified by scavenger receptors, leading to elevated uptakes of Ox-LDL by macrophages and the formation of foam cells. Previous studies were able to identify HNE-adducts in human atherosclerosis lesions by using anti-HNE antibodies [63,64]. The role of HNE in ACS has not been studied well; however, a study by Gargiulo et al. (2015) showed that HNE may induce plaque instability by increasing the expression and synthesis of inflammatory cytokines such as interlukine-8 (IL-8), interlukine1-β (IL-1β), tumor necrosis factor-α (TNF-α), and matrix metalloproteinase-9 (MMP-9) via Toll-like Receptors 4/Nuclear Factor-κB (TLR4/NF-κB) signaling pathways [65]. In addition, a recent study showed that levels of HNE in coronary sinus were significantly higher in STEMI patients before and after percutaneous coronary intervention (PCI) when compared with patients with stable ischemic heart disease (IHD) who underwent elective PCI [33]. 

In the last 30 years, numerous studies have extensively shown that elevated levels of MDA are associated with CVD. Having traditional CVD risk factors such as cigarette smoking [21,66], hypertension [66], hyperlipidemia [67,68], and diabetes [69,70] were reported to be significantly correlated with higher MDA levels. Increased levels of MDA have been reported in plasma of patients with atherosclerotic diseases [71]. A nested case-control cohort showed that LDL-MDA was a strong predictor of carotid wall thickness in hypercholesterolemic men [72]. In a perspective study with 634 patients having CVD, serum levels of MDA were strong predictors of cardiovascular events (including MI, stroke, hospitalizations for non-fatal cardiovascular events mainly UA), and major vascular procedures (percutaneous transluminal coronary angioplasty (PTCA)/coronary artery bypass grafting (CABG)), independent of traditional risk factors such as blood pressure (BP), total cholesterol, high-density lipoprotein-cholesterol (HDL-cholesterol), LDL-cholesterol, triglycerides (TG), age, gender, body mass index (BMI), and inflammatory markers in patients with coronary heart disease (CHD) [73]. In a study by Bagatini et al. (2011), increased levels of MDA were observed in MI patients and subjects with CVD risk factors (including cigarette smoking, hypertension, and family history of CHD) when compared to healthy controls [74]. 

### 4.2. OxPL 

Atherogenicity of OxPLs was shown by Hörkkö et al. (1999) as they found that OxPLs contribute to Ox-LDL recognition by macrophages. They also found that the monoclonal antibody E06, which binds to the phosphocholine head group of PLs on Ox-LDL, inhibits Ox-LDL uptakes by macrophages [75]. Since then, several studies have shown that OxPLs may have roles in various steps of atherosclerosis such as facilitating Ox-LDL uptake by macrophages [76], mediating cellular inflammatory responses [77], and stimulating angiogenesis [25]. 

1-palmitoyl-2-oxovaleroyl-sn-glycero-3-phosphorylcholine (POVPC), and 1-palmitoyl-2-glutaroyl-sn-glycero-3-phosphorylcholine (PGPC), which are derived from arachidonyl phosphatidylecholines, are produced during Ox-LDL modification and have been identified in atherosclerotic plaques [78,79]. These fragmented OxPLs are toxic and create tissue injury through inflammatory responses [77] and apoptosis [80]. LP (a) is the main carrier of OxPLs in plasma, although they can also be transferred by LDL and HDL [81]. Previous studies have demonstrated that levels of OxPLs are strongly correlated with LP (a) levels and the extent of coronary stenosis [82,83]. Therefore, it has been suggested that the atherogenicity of LP (a) can be attributed to OxPLs as its a carrier of proinflammatory oxidized molecules. 

Tsimikas et al. (2003) developed a method to measure OxPL by using murine monoclonal antibody E06 [13]. This antibody binds to the phosphorylcholine (PC) head group of OxPL, particularly POVPC. Therefore, the amount of PC-OxPL per apoB-100 (OxPL/ApoB) containing lipoproteins can be calculated. By using this approach, they showed that OxPL levels increased significantly in MI patients after PCI, suggesting that these compounds are released and/or generated as a result of plaque rupture [13,84]. Prospective studies have shown that OxPLs levels can be considered as biomarkers of atherosclerosis progression, cardiovascular death, MI, and stroke. In a prospective Bruneck study, the 5-year follow-up of 700 participants aged 40 to 79 years old showed that OxPLs levels were strongly and significantly associated with the presence, extent, and development of carotid and femoral atherosclerosis, and predicted the presence of symptomatic CVD [85]. The ten-year follow-up of this population showed that risk of cardiovascular events, which was defined as cardiovascular death, MI, stroke, and transient ischemic attack (TIA), were significantly elevated in participants in the highest tertile of OxPLs/apoB than those in the lowest tertile independent of traditional risk factors, suggesting that OxPLs/apoB levels may predict the risk of 10-year CVD events [86].

### 4.3. Oxylipins 

Pioneering studies have shown the association between oxylipins derived from AA with UA and atherosclerosis. Elevated levels of TXB2 have been reported in the coronary circulation of patients with unstable angina [87,88]. Moreover, Mallat Z et al. (1999) showed that HETEs levels were significantly higher in plaques obtained from symptomatic patients (with unstable plaque) versus patients with stable plaques [89]. Similarly, Waddington et al. (2001) found higher levels of 15-HETE and 11-HETE in atherosclerotic plaque retrieved from individuals undergoing carotid endarterectomy [90]. Recently, a targeted metabolomics study showed that among all identified metabolites, 20-HETE was the only compound that was significantly higher in patients with atheroma plaque when compared with healthy subjects [91].

New studies have also investigated the role of oxylipins in the diagnosis and prognosis of ACS and MI. A retrospective nested case-control study, comprised of 470 ACS patients and 39 subjects without CHD as a control group, was conducted in a Chinese population [15]. Among the ACS patients, subjects who had had a major adverse cardiovascular event (MACE) during the 1037 days of follow up period were identified as the MACE group, and ACS patients without MACE during this period were named as the non-MACE group. In this study, LTB4, 8-HETE, 11-HETE, 12-HETE, and 15-HETE were significantly elevated in the ACS patients (both the MACE and non- MACE groups) when compared with the controls. In addition, the levels of 5-HETE and 9-HETE were significantly higher in the MACE group when compared with the controls, suggesting the potential diagnostic value of these oxylipins in ACS. In addition, the levels of 20-HETE were significantly elevated in the STEMI group when compared with the non-STEMI group, indicating that the pathogenesis of STEMI and non-STEMI may be different. Moreover, the 19-HETE levels, a vasodilator oxylipin, were significantly lower in the MACE group than the non-MACE and control groups. ACS patients who had higher levels of 19-HETE (higher than 0.13 ng/mL) tended to have better prognosis (up to 72%) than those with lower levels [15]. In a prospective study by Sun et al. (2016) [92], the association between oxylipins and the incidence of MI was investigated in 744 AMI cases and 744 matched controls, aged 47–83 years within the Singapore Chinese Health Study. They found inverse correlations between pro-thrombotic TXB2 and AMI risk, and suggested that this unexpected association was more related to sample collection, processing, and storage conditions than biological differences. Moreover, in this study, only 19 oxylipins, which had potential roles in inflammation, blood pressure, and platelet degranulation were measured, and not the full spectrum. In a study by Caligiuri et al. (2017), the associations between oxylipins and the occurrence of cardiovascular/cerebrovascular events, defined as STEMI, non-STEMI, and UA, in 24 patients with peripheral artery disease (PAD) were assessed. They found that levels of 16-HETE, TXB2, and 11,12-DiHETrE were significantly associated with increased odds of cardiovascular/cerebrovascular events in PAD patients and showed that with every 1 nmol/L increase in 8,9-DiHETrE concentrations, the odds of ACS increased by 454-fold. In this particular study, 8,9-DiHETrE elevated the odds of ACS by 92-fold [18].

All of the clinical studies that have assessed these bioactive lipids in ACS patients are presented in Table 1.

## 5. Conclusions

There is accumulating evidence that bioactive lipids play roles in ischemic cardiovascular disease. We have made great strides in elucidating their activity by utilizing antibody based approaches. Given the advances in mass spectrometry, we were able to identify and quantitate individual oxidized lipids in plasma. It is important that we standardize the current mass spectrometric methods of quantitation and analysis, so that large cohorts of patients can be analyzed. This would lead to a better understanding of the specific contribution of each lipid molecule to the overall pathophysiology.

## Figures and Tables

**Table 1:** Clinical studies that have assessed bioactive lipids in ACS patients

Oxidized Lipids	Author	Year	Method of Detection	Population	Results
**HNE/MDA**	Aznar [4]	1983	TBARS-spectrophotometry	MI patients, patients with angina pectoris (AP), and normal control group	MDA values were normal in AP patients. MDA levels increased significantly following MI and reached maximum levels in 6–8 days
	De Scheerder [32]	1991	TBARS-spectrophotometry	CABG surgery candidates	Levels of MDA increased after repetitive coronary occlusions during coronary angioplasty. After 5-min of reperfusion, MAD levels further increased. Following 15-min of reperfusion, it returned to baseline levels
	Walter [73]	2004	HPLC-spectrophotometry	Patients with documented CAD	Baseline levels of MDA were higher in patients who had major/nonfatal MI, and major vascular procedures after three-year study
	Kaminski [33]	2009	HPLC-spectrophotometry	STEMI patient and stable angina patients (as controls)	Higher HNE and MDA levels in STEMI patients compared to controls
	More [14]	2017	TBARS-spectrophotometry	MI patients and normal healthy control	Higher MDA levels in MI patients compared to control
	Ismail [12]	2018	TBARS-spectrophotometry	MI patients and healthy controls	Higher MDA levels in MI patients compared to control
**OxPL**	Tsimikas [13]	2003	OxPL/ApoB	Patients with ACS (MI and unstable angina), stable angina and healthy subjects	Baseline levels of OxPL/ApoB were significantly higher in ACS patients compared with stable angina and healthy controls. In MI patients, OxPL/ApoB increased by 54% and 36% at hospital discharge and 30 days, respectively
	Tsimikas [84]	2004	OxPL/ApoB	Patients with stable angina pectoris undergoing PCI	OxPL/ApoB levels increased following PCI
	Tsimikas [83]	2005	OxPL/ApoB	CAD patients underwent coronary angiography	Percentage of stenosis was correlated with OxPL/apoB levels. OxPL/apoB levels predicted CAD independent of all other clinical markers except for LP (a)
	Tsimikas [86]	2006	OxPL/ApoB	Subjects aged 40 to 79 year-old followed for 5 years	OxPL/ApoB levels predicted the presence of symptomatic CVD
	Kaminski [33]	2007	OxPL/ApoB	Subjects aged 40 to 79 year-old followed for 10 years	OxPL/ApoB levels predicted future cardiovascular events independent of FRS
	Byun [16]	2015	OxPL/ApoB	Patients treated with intensive versus moderate atorvastatin therapy: the TNT trial	OxPL/apoB levels predicted secondary MACE
	Leibundgut [93]	2016	OxPL/plasminogen (PLG) and plasminogen	Patients with stable angina	OxPL/PLG and plasminogen decreased significantly immediately after PCI, rebounded to baseline at 6 h post-PCI, peaked at 3 days and slowly returned to baseline by 6 months
	Byun [17]	2017	OxPL/ApoB	Patients with prior stroke or TIA	Elevated baseline levels of OxPL/apoB predicted recurrent stroke and first major coronary events after five-year follow up
**Oxylipins**	Strassburg [58]	2012	HPLC-MS	Patient underwent cardiac surgery	Increased levels of 12-HETE and 12-HEPE at 24 h-post cardiac surgery
	Zu [15]	2016	UPLC-MS	ACS patients with or without MACE during follow up	20-HETE level was significantly higher in STEMI group comparing with NSTEMI. ACS patients with 19-HETE levels tended to have better prognosis for MACE.
	Auguet [91]	2018	HPLC-MS	Patients who underwent carotid endarterectomy	20-HETE levels were significantly higher in patients with atheroma plaque than healthy subjects
	Caligiuri [18]	2017	HPLC-MS	Patients with PAD	8,9-DiHETrE increased the odds of ACS. A positive relationship was observed between plasma concentrations of 18-HEPE and ACS.
